# Supplementary material for: Dynalign II: common secondary structure prediction for RNA homologs with domain insertions
Source: Nucleic Acids Res. 2014 Nov 21;42(22):13939–48. doi: 10.1093/nar/gku1172 (PMC4267632; doi:10.1093/nar/gku1172)
Supplement: SUPPLEMENTARY DATA [file supp_gku1172_nar-02021-z-2014-File012.zip › manual/javadoc/RNAstructure_java_utilities/package-summary.html]

RNAstructure\_java\_utilities


JavaScript is disabled on your browser.


- Overview
- Package
- Class
- Tree
- Index
- Help

- Prev Package
- Next Package

- Frames
- No Frames

- All Classes

# Package RNAstructure\_java\_utilities

A package that holds a variety of utilities classes.

See: Description

- Class Summary

  | Class | Description |
  | --- | --- |
  |  |  |
  | --- | --- |
  | BorderBuilder | A class that places a border type on a specified component type. |
  | BorderedButtonPanel | A class that produces a panel which contains a button with a border around it. |
  | FieldPanel | A class that creates a panel which holds aligned input fields. |
  | FieldPanel.FilePanel | An inner class that handles a panel which contains file fields. |
  | FieldPanel.NumberPanel | An inner class that handles a panel which contains numeric data fields. |
  | FileChooser | A class that creates a file chooser so the user can select a file to open or save. |
  | FileField | A class that creates a text field which holds a file name. |
  | HTMLCheckBox | A class that creates a check box whose text is HTML. |
  | ImageGrabber | A class that creates images in various contexts for the RNAstructure GUI. |
  | MacChecker | A class that handles checking whether an OS is a Mac, and setting general properties if that is the case. |
  | NumberField | A class that creates a text field whose contents may be bounded by a minimum or maximum value. |
  | NumberField.DoubleField | An inner class that handles a field which contains a double value. |
  | NumberField.FloatField | An inner class that handles a field which contains a float value. |
  | NumberField.IntegerField | An inner class that handles a field which contains an integer value. |
  | RadioButtonPanel | A class that creates a panel containing a group of radio buttons. |
  | RNAstructureMenu | A class that creates a menu. |
  | ScrollerPane | A class that creates a scroll pane that can be controlled via the keyboard. |
  | SimpleDialogHandler | A class that handles simple dialogs used in the RNAstructure GUI. |
  | ValueSelectionDialog | A class which creates a dialog that selects values from labeled text fields. |
- Enum Summary

  | Enum | Description |
  | --- | --- |
  |  |  |
  | --- | --- |
  | FileChooser.Types | An enum that holds the possible values for the file chooser type. |


## Package RNAstructure\_java\_utilities Description

A package that holds a variety of utilities classes.
  
These utilities classes encapsulate complex objects or functionalities that are used in many places.

- Overview
- Package
- Class
- Tree
- Index
- Help

- Prev Package
- Next Package

- Frames
- No Frames

- All Classes
